# Study on Neuroendocrine-Immune Function of *Cistanche deserticola* and Its Rice Wine Steaming Products in Glucocorticoid-Induced Rat Model

**DOI:** 10.1155/2020/5321976

**Published:** 2020-12-22

**Authors:** Bonan Liu, Ji Shi, Zhe Li, Chao Zhang, Pengpeng Liu, Wei Yao, Tianzhu Jia

**Affiliations:** ^1^School of Pharmacy, Liaoning University of Traditional Chinese Medicine, Dalian 116600, China; ^2^The Second Hospital of Dalian Medical University, Dalian 116600, China

## Abstract

The desert-dwelling *Cistanche* herb was first recorded in the “Shen Nong Herbal Classic” and is listed as the top-grade herbal medicine in this publication. The Chinese Pharmacopoeia records that pieces of *Cistanche deserticola* (CD) and rice wine-steamed *Cistanche deserticola* (WCD) can be used in the clinic as the main types of decoctions. After being steamed with rice wine, the antiaging and tonifying kidney-yang effects are enhanced. In this study, we detected the chemical content of CD and WCD and the pharmacological mechanism of invigorating kidney-yang deficiency in model rats. *Aim*. The purpose of this study was to examine the effects of CD and WCD on the neuroendocrine-immune function of kidney-yang deficiency in glucocorticoid-overdosed model rats. *Materials and methods*. Sprague Dawley (SD) rats were selected. The rats were subcutaneously injected with corticosterone water suspension for the glucocorticoid-overdosed model rats. The positive control rats were gavaged with Jinkuishenqi pills and high-, medium-, and low-dose CD/WCD suspensions (1.646 g/(kg day), 5.48 g/(kg day), 2.74 g/(kg day), and 1.37 g/(kg day), respectively); the blank control (BC) and model control (MC) groups were given the same volume of distilled water as those in the drug group for 40 consecutive days at a dose of 1 mL/100 g. After the last administration, the blood was collected from the abdominal aorta, and serum levels of T, CRH, ACTH, CORT, cortisol, IL-10, IL-6, IL-2, TNF-*α*, and IFN-*γ* were measured. Organ indexes of the thymus gland and the spleen were calculated. The expression of Bax, Bcl-2, caspase-3, Fas, and FasL in the adrenal gland was measured by immunohistochemistry. The pathological changes in the thymus gland and the adrenal gland were observed by HE staining (×200). T lymphocyte subsets in peripheral blood were detected by flow cytometry, and the expression of CaM mRNA in the hypothalamus and hypophysis tissues was also measured by RT-PCR. *Results*. Compared with the MC group, the CD and WCD groups exhibited increases in activity, the organ index of the thymus and the spleen, the serum levels of T, CRH, ACTH, CORT, cortisol, IL-2, and IL-10, the ratio of CD4+/CD8+, and the expression of Bcl-2, caspase-3, Fas, FasL, and CaM in the hypophysis tissue. The CD and WCD groups also exhibited reductions in the IL-6, TNF-*α*, and IFN-*γ* levels in serum and the expression of CaM mRNA in the hypothalamus. *Conclusions*. Each dose of CD and WCD could counteract the dysregulated sex hormone and immune factors in glucocorticoid-overdosed model rats, enhancing and restoring the effect of the hypothalamic nerve cells and improving immune function.

## 1. Introduction

*Cistanche deserticola*, which is called “desert ginseng,” is a traditional Chinese medicine that has been used for centuries as a yang-tonic herb for invigorating the kidney and strengthening the yang [1]. Eight species and one variation of *Cistanches Herba* have been recorded in China and only *Cistanche deserticola* Y. C. Ma and *Cistanche tubulosa* (Schenk) Wight are recorded in the Chinese Pharmacopoeia [2]. Pharmacological studies demonstrated that *Cistanches Herba* exhibited neuroprotective [3], immunomodulatory [4], cardioprotective [5], antifatigue [6], anti-inflammatory [7], hepatoprotective [8], antioxidative [9], antibacterial [10], laxative [11], and antitumor effects [12]. The broad spectrum of reported biological activities of this genus has been attributed to the complex and varied phytochemical composition. *Cistanche deserticola* contains phenylethanol glycosides, iridoids, polysaccharides [13], etc. The content of phenylethanoid glycosides is the highest in the original medicinal materials [14].

Processing of Chinese Materia Medica (CMM) is a traditional pharmaceutical technique to fulfill the different requirements for treating, dispensing, and making preparations according to traditional Chinese medicine theory. Those processed products are named as decoction pieces, which are used in clinics. The aims of processing are to enhance the efficacy and/or reduce the toxicity of crude drugs. 2015 Chinese Pharmacopoeia recorded that *Cistanche thick slices* (CD) and *rice wine-steamed Cistanche* (WCD) could be used as decoction pieces in the clinic. Our research group showed that the laxative effect was relieved, and the antiaging and kidney-yang invigorating effects were enhanced after *Cistanche* was steamed with rice wine [15].

The function of the hypothalamus-pituitary-adrenal gland-thymus gland (HPAT) axis is disordered in the kidney-yang deficiency syndrome [16]. Patients with kidney-yang deficiency also have extensive immune dysfunction. HPA axis dysfunction is closely related to immunodeficiency. Neuroendocrine-immune network (NEI) theory shows that the immune and neuroendocrine systems share many ligands and receptors [17], and the hypothalamus is considered the pivot to link the neuroendocrine with the immune systems.

In this study, we replicated kidney-yang deficiency in model rats with exogenous corticosteroid injection and explored the mechanism of the kidney-yang deficiency in response to different processed products of *Cistanche deserticola* from the perspective of endocrine-immune function.

## 2. Materials and Methods

### 2.1. Materials and Reagents

*Cistanche deserticola* was collected from Alashan Neimenggu in 2019.5 and identified as the dried fleshy stems of *Cistanche deserticola* Y. C. Ma by Prof. Yanjun Zhai (*School of Pharmacy, Liaoning University of Traditional Chinese Medicine, China*). The voucher specimens were preserved in the Liaoning Processing Engineering Technology Center.

Standard compounds of ajugol were purchased from Chengdu Pure Chem-Standard Co., Ltd. (Chengdu, China); cistanoside F, echinacoside, cistanoside A, and isoacteoside were purchased from Must Company (Sichuan China); acteoside was purchased from Dalian Meilun Bio. Co., Ltd. (Dalian, China). MS-grade acetonitrile and methanol were purchased from Merck KGaA (Darmstadt, Germany). HPLC-grade formic acid was purchased from Merck KGaA (Darmstadt, Germany). Rice wine was purchased from Zhejiang Brand Tower Shaoxing Wine Co., Ltd. (Zhejiang, China).

Corticosterone (CAS: 50-22-6, purity > 98%, TCI Shanghai Development Ltd. Co.), chinkuei shin chewan pills (Beijing Tongrentang Technology Development Co., Ltd.), rat ACTH, CRH, T, IL-6, IFN-*γ*, TNF-*α*, IL-2, and IL-10 ELISA detection kits were purchased from Nanjing Jiancheng Co., Ltd. The rat cortisol ELISA kit was provided by BOSK Co., rat CD4 antibody, CD8a antibody (nos. 561833 and 559976), RBC lysate (Solarbio, R1010), PBS buffer solution (Solarbio, P1020-500), TRIzol (MAN0001271), CaM, and *β*-actin upstream and downstream primers were provided by the Guangzhou Invitrogen Co. Reverse Transcription Kit (no. RR037A) and cDNA Synthesis Kit (no. 10000068167) were provided by Bole Life Medicine Products (Shanghai) Co., Ltd. Chloroform, isopropanol, 75% ethanol, and urethane solution were all analytically pure and produced by Sinopharm Chemical Reagent Co., Ltd. Ultrapure water was produced by the Milli-Q system (18.2 MΩ, Millipore, MA, USA).

### 2.2. Experimental Instruments

An enzyme marker (Thermo, model 3530911931), automatic plate washer (model HBS-4009), digital display push-pull force meter (model VICTOR-50N), high-speed freezing centrifuge (model Sigma 3k15), ultramicrospectrophotometer (model B-50Q), gene amplifier (model L96G), real-time fluorescence quantitative PCR (model StepOne), flow cytometer (model AC66051710132), paraffin microtome (model Laika), microscope (Olympus, model BS-53), and a pure water instrument (model F7JA36507) were used.

### 2.3. Preparation of Samples for UPLC-Q-TOF-MS and Pharmacological Experiments

WCD was processed from the same batch of *Cistanche deserticola* in our laboratory. For the preparation of WCD, dry CD pieces (5 mm thick) (100 g) were infused with rice wine (30 mL), steamed at high pressure (1.25 kPa) for 4 h, and then dried at 55°C.

The coarse powders of CD and WCD were soaked in 10 times 95% ethanol for 0.5 h, reflux-extracted 3 times each time for 1 h, and filtered, and the filtrates of 3 times were combined. The ethanol was recovered under reduced pressure, and the extract was obtained for administration. The extract (1 mL) was added to 50% methanol, bringing the total volume to 20 mL, and stored at 4°C for content analysis.

### 2.4. UPLC-QqQ-MS Conditions for Analysis of CD and WCD Extracts

#### 2.4.1. LC/MS Analytical Conditions

The UPLC-MS/MS system with MassLynx 4.1 Analyst software was used to perform the data acquisition and processing. The analytical column was an Acquity UPLC BEH C_18_ (100 mm × 2.1 mm, 1.7 *μ*m) at a temperature of 40°C. The mobile phase was 0.1% formic acid aqueous solution (A) and acetonitrile containing 0.1% formic acid (B). The elution gradient was 0.00–1.00 min with 3% B, 1.01–2.00 min with 3%–11.5% B, 2.01–3.00 min with 12% B, 3.01–4.00 min with 15% B, 4.01–5.00 min with 20% B, 5.01–6.00 min with 22% B, 6.01–8.00 min with 25% B, and 8.01–9.00 min with 10% B. The flow rate was 0.3 mL/min, and the injection volume was 1.0 *μ*L.

The Waters triple quadruple mass spectrometer (Xevo TQD, Waters Corp., Milford, MA, USA) equipped with an electrospray ionization (ESI) source was used in the negative ion mode. The desolvation gas was nitrogen with a flow rate of 500 L/h at a temperature of 250°C. All detected compounds were measured in the multiple reaction monitoring (MRM) mode; Table 1 shows the energy parameters, and Table 2 shows the calibration curves for analyzed components.

#### 2.4.2. Preparation of Reference Substances

Tubuloside A (3.02 mg), echinacoside (3.00 mg), 2′-acetylacteoside (2.34 mg), acteoside (2.45 mg), isoacteoside (0.61 mg), cistanoside F (2.14 mg), salidroside (3.39 mg), geniposidic acid (2.84 mg), ajugol (1.58 mg), and catalpol (2.39 mg) were dissolved in methanol to prepare a stock solution. The stock solution was diluted with methanol to get the appropriate concentrations for the working standard solutions. All prepared solutions were stored at 4°C before use.

### 2.5. Preparation and Grouping of Animal Models

SD male rats (180–220 g) were purchased from Liaoning Changsheng Biotechnological Co., Animal permit number: SCXK (Liao) 2015–0001. All rats were maintained with free access to food and water at 25°C and a relative humidity of 30–50%. The animals were housed for 7 days prior to the experiments.

One hundred rats were randomly divided into 10 groups: blank control (BC) group, model control (MC) group, positive control (PC) group, rice wine control (WC) group, CD high-dose (CD-HD) group, CD middle-dose (CD-MD) group, CD low-dose (CD-LD) group, WCD high-dose (WCD-HD) group, WCD middle-dose (WCD-MD) group, and WCD low-dose (WCD-LD) group. The doses of CD-HD/WCD-HD, CD-MD/WCD-MD, and CD-LD/WCD-LD were 5.48 g/(kg ∙ d), 2.74 g/(kg ∙ d), and 1.37 g/(kg ∙ d), respectively. The dose for the PC group was 1.646 g/(kg ∙ d), and for the WC group, 1 mL/100 g for 40 days. The 6^th^ day after administration, the rats were subcutaneously injected with the corticosterone water suspension (corticosterone + 0.1% dimethyl sulfoxide + 0.1% Tween-80 + 0.9% sodium chloride) except for the BC group [18]. The concentration of corticosterone was 5 g/L, and the dose was 0.1 mL/100 g. The rats in the BC group were given normal saline + 0.1% dimethyl sulfoxide + 0.1% Tween-80 + 0.9% sodium chloride by injection at the same dose.

### 2.6. Determination of HPA Axis Functions

#### 2.6.1. Weight, Temperature, and Holding Power Test

A weight test was performed every 3 days, and the temperature was taken every 6 days. during the experiment. On the 39^th^ day, the hind limb maximum strength was measured by a grip meter. The rats were placed on smooth platforms, making their hind limb grab the pole. The rats will instinctively grab any object to prevent moving backward when the tail is pulled, until the pull force exceeds the grip. When the rat lost its grip, the preamplifier could automatically record the maximum grip force.

#### 2.6.2. Determination of the Level of T, CRH, ACTH, CORT, and Cortisol in Serum

One hour after the last administration, the rats were anesthetized by intraperitoneal injection of a urethane solution (20 g/100 mL). Then, the blood samples were collected, centrifuged at 3500 r for 15 min to obtain the serum, and stored at −20°C. The concentrations of T, CRH, ACTH, CORT, and cortisol were measured with rat ELISA kits according to the manufacturer's instructions.

#### 2.6.3. Microscope Observations

The morphological structure of adrenal gland tissue was observed by HE staining. Adrenal tissue was fixed in 10% paraformaldehyde, dehydrated in ethanol, made transparent by a xylene solution, embedded by conventional methods, cut to 4 *μ*m thick slices, dewaxed with xylene solution, hydrated with the ethanol gradient, and then stained with hematoxylin and eosin staining solution. After being made transparent with xylene, the plate was sealed with neutral gum and observed under a microscope.

#### 2.6.4. Immunohistochemical Staining of Bax, Bcl-2, Caspase-3, Fas, and FasL Protein Expression in the Adrenal Gland

Formalin-fixed, paraffin-embedded rat adrenal sections were deparaffinized and rehydrated after being cut at a thickness of 4 *μ*m. For the blocking of endogenous peroxidase activity, the sections were preincubated in hydrogen peroxide block for 10 min. Sections were dipped into 0.01 M citrate (pH 6.0) and heated to boiling. The process was repeated after 5–10 min. After cooling, the sections were washed with PBS (pH 7.2–7.6) 1–2 times, left at room temperature for approximately 5 min, and washed with PBS (pH 7.2–7.6) 2–3 times. The 5% BSA blocking solution was added at room temperature, and the excess liquid on the section was shaken off 20 min later. Diluted primary antibodies specific for Fas/FasL, Bcl-2/Bax, and caspase-3 (1 : 100 diluted with PBS) were added and incubated at 37°C for 1 h or 4°C overnight. The sections were washed 2–3 times with PBS (pH 7.2–7.6). Then, biotinylated goat anti-mouse IgG was added, and the sections were dried at 20–37°C for 20 min and washed with PBS (pH 7.2–7.6) 4 times for 5 min. After thorough washing in PBS, the sections were incubated with a mixture of reagents A and B for 30 min, incubated with PBS for 45 min, and finally developed with DAB substrate (DAKO) for 30 min before being slightly counterstained with hematoxylin, dehydrated, and mounted.

### 2.7. Determination of Immune Functions

#### 2.7.1. Calculation of Immune Organ Index

One hour after the last administration, the rats were anesthetized by intraperitoneal injection of urethane solution (20 g/100 mL), and then the spleen and thymus gland were removed and weighed immediately in a sterile hood. The weight coefficients of the spleen or the thymus (%) = spleen or thymus weight (mg)/body weight (g).

#### 2.7.2. Detection of T Lymphocyte Subsets in Peripheral Blood

Splenocytes and hemocytes were incubated with the monoclonal antibodies anti-CD4 (PE) and anti-CD8 (FITC) for 30 min in the dark. 2 mL of erythrocyte lysate was added, and the solution was mixed by vortexing. The solution was left in darkness for 10 min and centrifuged for 5 min. The supernatant was discarded, and then the solution was washed 3 times with ice-cold PBS and resuspended in PBS permeabilizing solution. At least 10,000 cells were analyzed by flow cytometry within 1 h of each Mab staining.

#### 2.7.3. Determination of the Level of IL-10, IL-6, IL-2, TNF-*α*, and IFN-*γ* in Serum

One hour after the last administration, the rats were anesthetized by intraperitoneal injection of urethane solution (20 g/100 mL), and blood was taken from the abdominal aorta. The blood was centrifuged at 3500 r for 15 min to obtain the serum and stored at −20°C. The concentrations of IL-10, IL-6, IL-2, TNF-*α*, and IFN-*γ* were detected with rat ELISA kits according to the manufacturer's instructions.

### 2.8. Expression of CaM in Hypothalamus and Hypophysis Tissues

Total RNA was extracted from the hypothalamus and hypophysis tissues by TRIzol. Reverse transcription was carried out on 10 *μ*L of total RNA according to the manufacturer's instructions. Equal amounts of cDNA were analyzed via qPCR in the presence of dsDNA-binding dye (Promega, USA) and the CFX 96 Real-time PCR System (Bio-Rad, USA) to look at the different genes under the conditions of initial activation at 95°C for 10 min, 40 cycles of denaturation at 95°C for 15 s, and annealing/extension at 60°C for 1 min.

Primers for CaM and *β*-actin are given in Table 1, and *β*-actin was used as an internal control. Each sample was normalized by using the difference in critical thresholds (Ct) between the target gene and *β*-actin. The following equation was used to describe the result: ΔΔCt = (Ct target gene − Ct *β*-actin gene) experimental group − (Ct target gene − Ct *β*-actin gene) control group. The mRNA levels of each sample were then compared, using the expression 2-ΔΔCt target gene. The results of each group were averaged (Table 3). 

### 2.9. Statistical Analysis

All data were analyzed using SPSS software (version 19.0, SPSS Institute Inc., Chicago, IL). Differences between groups were analyzed with one-way repeated-measures analysis of variance (ANOVA). The results were expressed as mean ± standard deviation (SD) using GraphPad Prism software (version 6.0, San Diego, CA, USA). Differences with a *P* value less than 0.05 were considered statistically significant.

## 3. Experimental Results

### 3.1. UPLC-QqQ-MS Analysis

**Figure 1:**
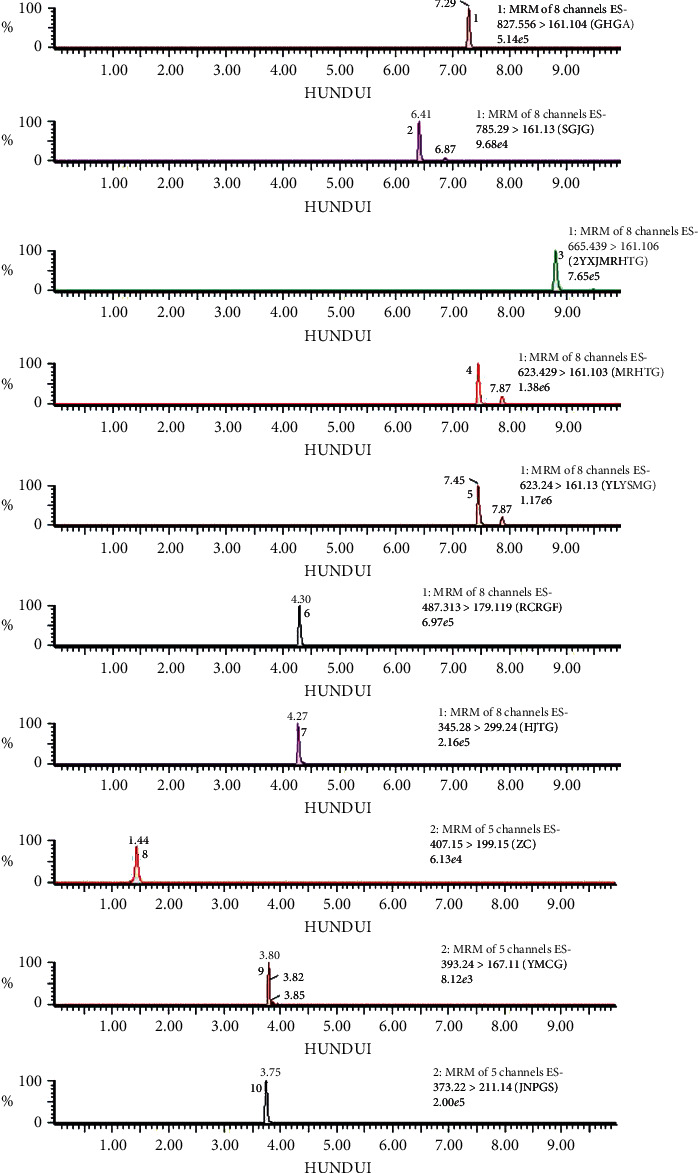
UPLC-QQQ-MS chromatogram of 10 components in CD and WCD: (1) tubuloside A; (2) echinacoside; (3) 2′-acetylacteoside; (4) acteoside; (5) isoacteoside; (6) cistanoside F; (7) salidroside; (8) catalpol; (9) ajugol; (10) geniposidic acid.

To achieve the best separation, peak shape, and a short analysis time, the chromatographic conditions including column, mobile phase, and gradient program were studied in our preliminary experiment. The typical chromatograms with MRM mode are presented in Figure 1.

From Table 4, we could see that the contents of phenylethanol glycosides in WCD increased compared to those of CD, especially for echinacoside and acteoside, while the content of iridoids was decreased in WCD.

### 3.2. Regulation for HPA Axis Function

#### 3.2.1. Weight, Temperature, and Holding Power Test

**Figure 2:**
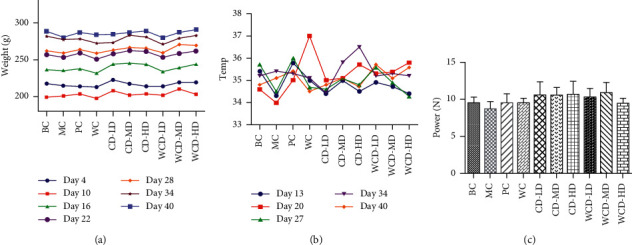
Change of body weight, body temperature, and holding power of rats. (a) Weight changing. (b) Temperature changing. (c) Holding power study.

The rats of the MC group and experimental groups showed kidney-yang deficiency symptoms gradually after being given corticosterone. The symptoms, such as weight loss, hypothermia, loss of hair luster, drooping spirit, lag in response, significant decrease in water consumption and activity, improved greatly in the CD and WCD groups.  Figure 2(a) shows the changes in weight. The weight of each of the drug groups increased, especially in the CD-HD and WCD-HD groups. The weight gain in the MC group was the lowest.  Figure 2(b) shows the changes in temperature, which was the lowest in the MC group, and the temperature increased in the WCD-HD, WCD-MD, and WCD-LD groups.

Figure 2(c) shows that the holding power increased for the BC group and each of the experimental groups, and the WCD-MD group was the highest, which demonstrated that signs of weakness induced by kidney-yang deficiency improved after administration.

#### 3.2.2. Levels of T, CRH, ACTH, CORT, and Cortisol

**Figure 3:**
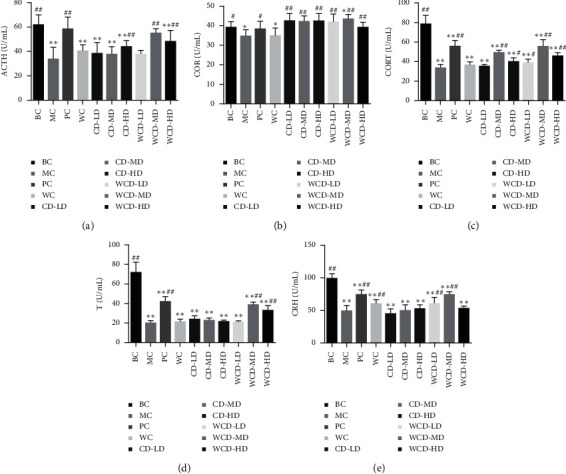
Effects on serum T, CRH, ACTH, CORT, and cortisol levels. Compared with the BC group, ^*∗*^*P* < 0.05, ^*∗∗*^*P* < 0.01; compared with the MC group, ^#^*P* < 0.05, ^##^*P* < 0.01 (*n* = 10 rats/group).

Figure 3 shows that compared to those of the BC group, the level of T, CRH, ACTH, and CORT in the rat serum decreased (*P* < 0.01) and cortisol level decreased (*P* < 0.05) in the MC group. Compared to those of the MC group, the level of T in the WCD-HD and WCD-MD groups increased (*P* < 0.01), level of CRH in the WCD-LD, WCD-MD, and WC groups improved (*P* < 0.01), content of ACTH increased in the CD-HD, WCD-MD, and WCD-HD groups (*P* < 0.01), CORT level upgraded in the CD-MD, WCD-MD, and WCD-HD groups (*P* < 0.01) and enhanced in the CD-HD and WCD-LD groups (*P* < 0.05), and cortisol level increased in each of the drug groups.

#### 3.2.3. Results of Microscope Observation

**Figure 4:**
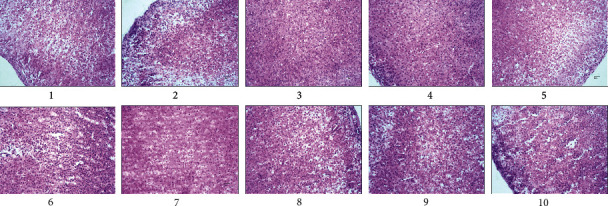
Pathological section of rat adrenal gland. (1) BC group; (2) MC group; (3) PC group; (4) WC group; (5) CD-LD group; (6) CD-MD group; (7) CD-HD group; (8) WCD-LD group; (9) WCD-MD group; (10) WCD-HD group.

Adrenal gland tissue can be divided into the cortical layer and the medulla layer. The cortical layers include globular, bundle, and reticular layers. The cells contain more lipids, and the medulla layer consists of mostly pheochromocytoma cells and a small amount of fibrous tissue. As shown in Figure 4, we found that all was normal in the BC group, while in the MC group, the adrenal cortex had obvious hyperplasia, cellular atrophy, and increasing density. The bulbous strip thickened, and the translucent fascicular zone, narrow zona reticularis, and smaller cells showed uneven coloring and capillary congestion. In the PC group, the cortical and medullar boundaries were clearly visible. The cells of the globular and bundle bands were arranged evenly, and the morphological structure had clearly been restored. The same better restoration was observed in the WCD groups, and the effects in the WCD-MD group were better than those of the WCD-HD and WCD-LD groups. The morphology of the adrenal gland in the CD groups was not as good as that in the WCD groups.

#### 3.2.4. Immunohistochemistry

Neuronal apoptosis is controlled by many apoptotic and antiapoptotic genes, mainly the apoptosis-promoting gene Bax and the antiapoptotic genes of the Bcl-2 gene family. Bcl-2 is localized in reactive oxygen-producing organelles such as the mitochondria, endoplasmic reticulum, and nuclear membranes. Bcl-2 has little impact on oxygen free radicals but can prevent oxidative damage in cells. Bax transports from the cytoplasm to the mitochondrial membrane, changes the mitochondrial membrane structure, and promotes the release of cytochrome c and the induction of apoptosis.

**Figure 5:**
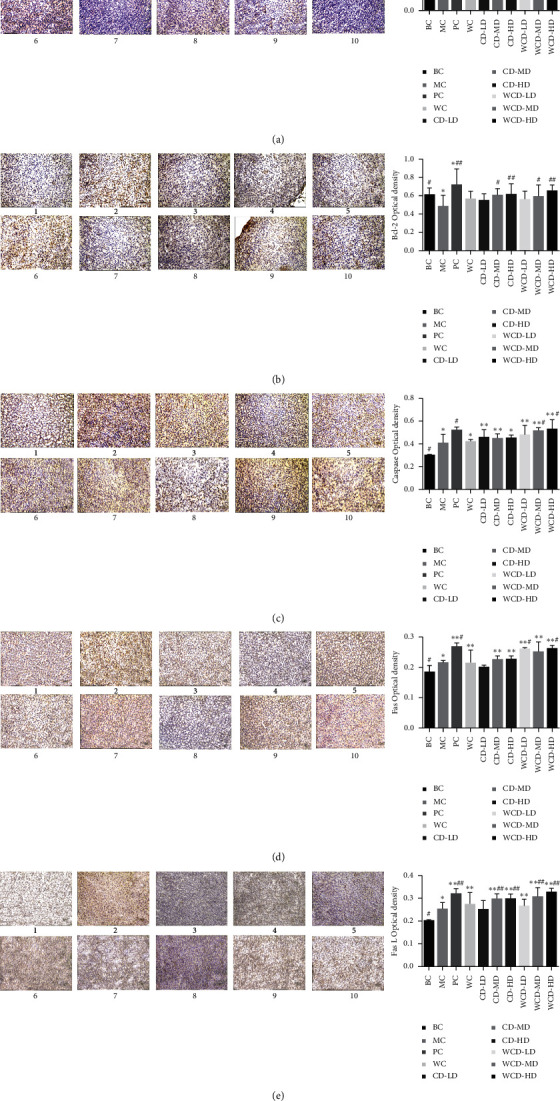
Immunohistochemical section of Bax gene in the adrenal gland. (a) Bax gene, (b) Bcl-2 gene, (c) Cas P gene, (d) Fas gene, and (e) FasL gene. (1) BC group; (2) MC group; (3) PC group; (4) WC group; (5) CD-LD group; (6) CD-MD group; (7) CD-HD group; (8) WCD-LD group; (9) WCD-MD group; (10) WCD-HD group. Compared with the BC group, ^*∗*^*P* < 0.05, ^*∗∗*^*P* < 0.01; compared with the MC group, ^#^*P* < 0.05, ^##^*P* < 0.01 (*n* = 10 rats/group).

There was expression in the adrenal cytoplasm of Bax and Bcl-2 protein. The staining showed all yellow particles. Compared to that of the BC group, the average optical density of the Bax protein in the MC group increased significantly (*P* < 0.01). Compared to that of the MC group, the average optical density in the CD-LD and WCD-LD groups decreased significantly (*P* < 0.01) (Figure 5(a)).

Figure 5(b) shows that compared to that of the BC group, the average optical density of Bcl-2 protein in the MC group decreased significantly (*P* < 0.05). Compared to that of the MC group, the average optical density in the WCD-HD and CD-HD groups increased significantly (*P* < 0.01), while those of the WCD-MD and CD-MD groups obviously increased (*P* < 0.05).

The caspase-3 protein is localized mainly in the cytoplasm in its original form in normal tissues and has low expression. Positive signals are pale yellow or brownish yellow. Compared to that of the BC group, the expression of caspase-3 in the MC group increased significantly (*P* < 0.05). Compared to that of the MC group, the expression of caspase-3 in the WCD-HD and WCD-MD groups obviously increased (*P* < 0.05) (Figure 5(c)).

In normal tissues, the expression of the Fas gene was reduced, and the positive signal for in situ hybridization was brownish yellow. As shown in Figure 5(d), compared to that of the BC group, the average optical density of Fas protein in the MC group obviously increased (*P* < 0.05). Compared to that of the MC group, the average optical density in the WCD-HD and WCD-LD groups obviously improved (*P* < 0.05) (Figure 5(d)).

The FasL protein is located mainly in the cell membrane or cytoplasm, and its positive signals are brown. Compared to that of the BC group, the average optical density of FasL gene in the MC group increased significantly (*P* < 0.05). Compared to that of the MC group, the average optical density of the FasL gene in the WCD-HD, WCD-MD, CD-HD, and CD-MD groups increased significantly (*P* < 0.01) (Figure 5(e)).

The results demonstrated that CD and WCD could induce apoptosis in inflammatory cells and reduce adrenal damage through promoting the expression of the FasL protein and binding to the Fas protein, activating caspase-3 protein and triggering a caspase cascade. The effect of WCD-HD group was the best.

### 3.3. Determination of Immune Function

#### 3.3.1. Immune Organ Index

**Figure 6:**
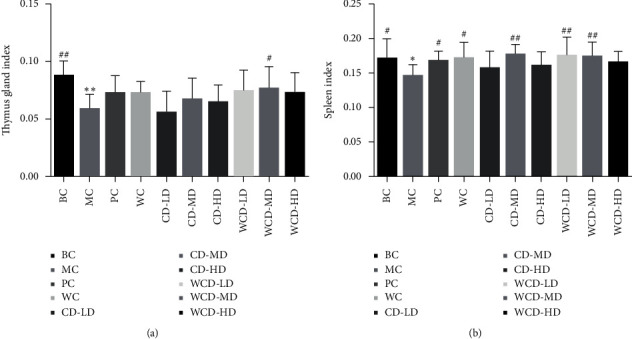
Changes of thymus index and spleen index in rats with low immune function. Compared with the blank control group, ^*∗*^*P* < 0.05, ^*∗∗*^*P* < 0.01; compared with the model control group, ^#^*P* < 0.05, ^##^*P* < 0.01 (*n* = 10 rats/group).

Compared to those of the BC group, the thymus coefficient and the spleen index of the MC group decreased significantly (*P* < 0.01). Compared to the MC group, the WCD-MD group had the greatest difference (*P* < 0.05). The thymus coefficient was almost back to the normal level. For the spleen index, the WCD-MD, WCD-LD, and CD-MD groups showed significant differences compared with that of the MC group (*P* < 0.01) (see Figure 6). Therefore, WCD and CD treatment could protect the immune organs, and the effects of WCD were better.

#### 3.3.2. Detection of T Lymphocyte Subsets in Peripheral Blood

**Figure 7:**
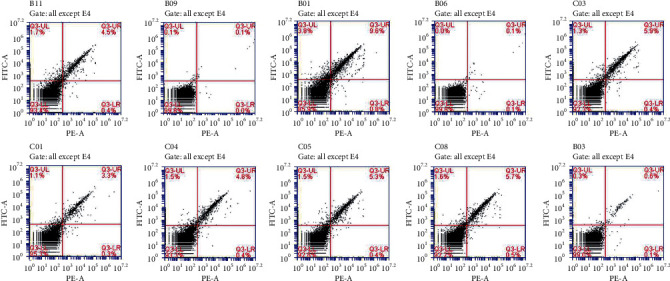
T lymphocyte flow cytometry in peripheral blood of rats. (1) BC group; (2) MC group; (3) PC group; (4) WC group; (5) CD-LD group; (6) CD-MD group; (7) CD-HD group; (8) WCD-LD group; (9) WCD-MD group; (10) WCD-HD group.

The percentage of CD4+ cells in the upper left quadrant and CD8+ cells in the right lower quadrant in the BC and each drug group was higher than that in the MC group. Compared to that of the BC group, the ratio of CD4+/CD8+ decreased significantly in the MC group (*P* < 0.01). Compared to that of the MC group, the ratio of CD4+/CD8+ increased significantly in the PC group, the WCD groups, and the CD-HD group (*P* < 0.01) (Figure 7).

#### 3.3.3. Determination of the Level of IL-10, IL-6, IL-2, TNF-*α*, and IFN-*γ* in Serum

**Figure 8:**
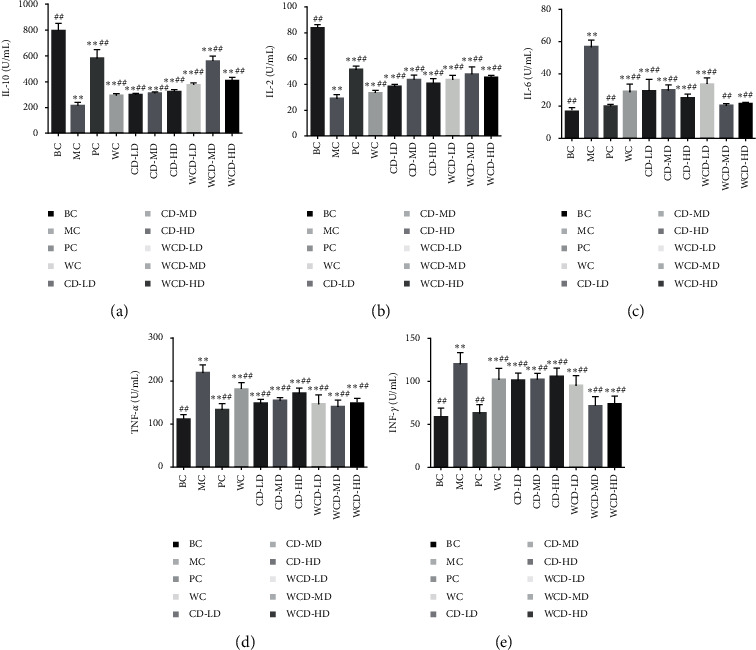
Level of IL-10, IL-6, IL-2, TNF-*α*, and IFN-*γ* in rat serum. Compared with the blank control group, ^*∗*^*P* < 0.05, ^*∗∗*^*P* < 0.01; compared with the model control group, ^#^*P* < 0.05, ^##^*P* < 0.01 (*n* = 10 rats/group).

Figure 8 shows that compared to that of the BC group, the level of IL-6, TNF-*α*, and IFN-*γ* increased significantly (*P* < 0.01). The level of IL-10 and IL-2 decreased in the MC group. Compared to that of the MC group, the level of IL-6, TNF-*α*, and IFN-*γ* decreased and IL-10 and IL-2 increased in serum in each of the drug groups. The effect of WCD was better than the effect of CD, especially in the WCD-MD group, in which the effect was close to that of the PC group.

#### 3.3.4. Results of Thymus Tissue Microscope Observation Experiment

**Figure 9:**
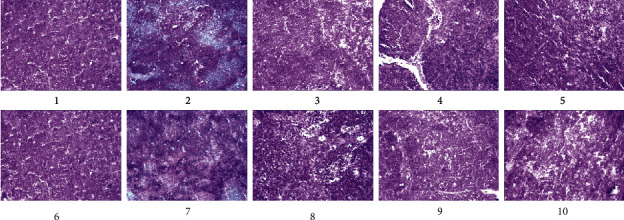
Pathological section of rat thymus. (1) BC group; (2) MC group; (3) PC group; (4) WC group; (5) CD-LD group; (6) CD-MD group; (7) CD-HD group; (8) WCD-LD group; (9) WCD-MD group; (10) WCD-HD group.

Figure 9 shows that in the BC group, a large number of lymphocytes were present. Lymphocytes and reticulocytes were loosely arranged in the center with scattered round or oval thymocytes. There was no necrosis, no inflammatory cell infiltration, and no abnormal changes.

The MC group showed a large amount of lymphoid necrosis tissue, with a disordered arrangement of lymphocytes and reticulocytes in the center. Round or oval thymocytes disappeared, massive cell degeneration or necrosis was evident, and there was inflammatory cell infiltration with abnormal pathological changes.

In the CD groups, the morphological characteristics of lymphocytes had returned to normal, and the arrangement of central lymphocytes and reticulocytes was almost normal. Round or oval thymocytes had recovered, but a small number of degenerative or necrotic lymphocytes and infiltrating inflammatory cells remained.

In the WCD groups and the PC group, the morphological characteristics of lymphocytes were better repaired. The lymphocytes and reticulocytes in the center were well aligned, the round or oval thymocytes were almost normal, but there remained a small number of infiltrating and degenerative lymphocytes.

### 3.4. CaM Protein Gene Expression

**Figure 10:**
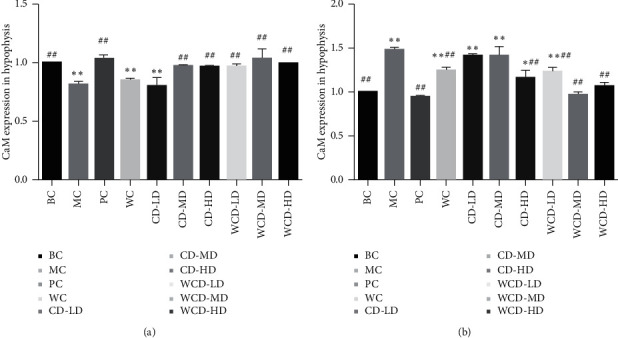
Results of CaM Gene Expression in Rats. Compared with the blank control group, ^*∗*^*P* < 0.05, ^*∗∗*^*P* < 0.01; compared with the model control group, ^#^*P* < 0.05, ^##^*P* < 0.01 (*n* = 10 rats/group).

RQ value (CaM mRNA/*β*-actin mRNA) was considered to examine the expression of CaM mRNA. The RQ of the BC group was 1. The expression of CaM mRNA in the hypothalamus in the MC group increased significantly (*P* < 0.01), while the value obviously decreased in the WCD-HD, WCD-MD, WCD-LD, and CD-HD groups (*P* < 0.05) (Figure 10). The expression of CaM mRNA in the hypophysis in the MC group was lower than that in the BC group (*P* < 0.01). The expression in the WCD-HD, WCD-MD, WCD-LD, CD-HD, and CD-LD groups obviously increased (*P* < 0.05), and the effect in the WCD groups was better than the effect in the CD groups.

## 4. Discussion

The Chinese Pharmacopoeia [2] records that CD and WCD can be used in the clinic as the main types of decocting pieces. After being steamed with rice wine, the antiaging and tonifying kidney-yang effects were enhanced. Through UPLC-QqQ-MS, we detected the quantitative changes in the chemical material base during the process of rice wine steaming. Iridoid glycosides were unstable and the content of these glycosides decreased, even becoming undetectable. The content of phenylethanol glycosides also changed, which could induce changes in the pharmacodynamics and the clinical effect.

The classic kidney-yang deficiency model was replicated by subcutaneous injection of corticosterone [19]. Overdose corticosterone could inhibit the secret of CRH in the hypothalamus. Meanwhile, the secret of ACTH was also inhibited. The level of adrenal cortex hormone decreased, and thus, the function of the HPA axis was reduced. Hypofunction of the HPA axis is a common characteristic of kidney-yang deficiency [20]. Then the rats show “depletion” phenomena, and the symptoms are in accord with those of kidney-yang deficiency in the clinic.

In the MC group, the level of CRH, ACTH, T, and CORT in rat serum decreased significantly (*P* < 0.01). After being treated by CD and WCD, the content of CRH, ACTH, T, CORT, and cortisol all increased to some degree, and the effect of WCD-MD was the best, almost equal to that in the PC group. These results indicated that the relative hormone balance in the HPA axis had been destroyed in the MC group, CD and WCD could improve the functional recovery of abnormal adrenal axis hormone levels, but the effect of WCD was better.

Modulating the proportion of Bcl-2 family members is one of the core mechanisms by which Fas/FasL mediates the death receptor pathway, and the Bcl-2 family regulates the mitochondrial pathway [21]. Apoptosis-inhibiting factor Bcl-2 and apoptotsis-inducing factors, such as Bax, all belong to the Bcl-2 family [22]. Cell apoptosis may be determined by the relative ratio of apoptosis-inhibiting factors and apoptosis-inducing factors [23]. Mitochondria had the function as central checkpoints for many forms of apoptosis [24]. The present study showed that CD-MD and WCDs could deduce the expression of Bax and increase Bcl-2 to improve the kidney-yang deficiency. Fas is the major death receptor that induces apoptosis by ligation with the Fas ligand (FasL) [25]. The expression of Fas and FasL in the MC group increased, while this expression increased significantly in the WCD-MD group (*P* < 0.01) compared with the MC group. Both Fas/FasL and Bax/Bcl-2 systems transduce apoptotic signal to the caspase family [26]. Caspase-3 is one of the pivotal proteinases that initiate cell apoptosis. The activation of caspase-3 indicates that apoptosis enters an irreversible stage [27]. The expression of caspase-3 in the MC group increased and increased significantly in the WCD-HD and WCD-MD groups (*P* < 0.05).

Deficiency of immune function is one of the important manifestations of kidney-yang deficiency [28]. In this study, we found that the index of the spleen and thymus of the WCD-MD group recovered significantly (*P* < 0.01, *P* < 0.05). The immune response of the immune system is regulated mainly by the Th subgroups [29]. IL-2 is the growth factor for T cells, secreted by T cells and macrophages, participates in inflammatory regulation, regulates immunity, and increases infection resistance [30]. The level of IL-2 in the MC group decreased (*P* < 0.01), and after treatment with CD and WCD, the level of IL-2 recovered. IL-6 and TNF-*α* are proinflammatory cytokines. Increased levels of IL-6 and TNF-*α* cytokines cause cytokine imbalances and exacerbate inflammation. The levels of IL-6 and TNF-*α* increased in the MC group, while after treatment, the levels of IL-6 and TNF-*α* decreased. T cells are divided into CD4 cells and CD8 cells, and the CD4+/CD8+ ratio can be used as an important index of immune regulation [31]. CD4+/CD8+ in the MC group obviously decreased (*P* < 0.01), while in the WCD and CD-HD groups increased (*P* < 0.01).

The HPA axis is the most important regulatory system because it regulates the glucocorticoid secretion and the endocrine system to maintain the secretory function of the adrenal cortex [32]. Overexpression of CaM mRNA in hypothalamus tissue was related to deficiency of kidney yang. CD and WCD could block the calcium channels, inhibit CaM mRNA expression in the hypothalamus, and decrease calcium influx, thus decreasing Ca2+·CaM complex activity.

The formation of kidney-yang deficiency has a chronic disease development process. Kidney is no longer a purely anatomical concept, and it is a general term for neuroendocrine-immunity and genitourinary system. Neuroendocrine-immune (NEI) network is an important integrated regulatory system [33]. CD and WCD can regulate the function of the HPAT axis and improve the recovery of kidney-yang deficiency. This result provides the experimental basis for the clinical prevention and treatment of kidney-yang deficiency.

## Figures and Tables

**Table 1:** The MS/MS transitions and energy parameters of analytes.

Compounds	Parent ion (*m*/*z*)	Daughter ion (*m*/*z*)	Voltage (V)	Collision energy (V)	Retention time (min)	ESI mode
Tubuloside A	827.56	161.1	82	46	7.29	ES^−^
Echinacoside	785.29	161.13	76	48	6.41	ES^−^
2′-Acetylacteoside	665.44	161.11	70	36	8.81	ES^−^
Acteoside	623.43	161.1	78	34	7.45	ES^−^
Isoacteoside	623.24	161.13	68	40	7.87	ES^−^
Cistanoside F	487.31	179.12	46	20	4.3	ES^−^
Salidroside	345.28	299.24	22	10	4.27	ES^−^
Geniposidic acid	373.22	211.14	38	10	3.75	ES^−^
Ajugol	393.24	167.11	26	12	3.8	ES^−^
Catalpol	407.15	199.15	24	12	1.44	ES^−^

**Table 2:** Calibration curves and LLOQs of components.

Standards	Calibration curves	*R* ^2^	Range (mg · mL^−1^)	LLOQ (*μ*g · mL^−1^)
Tubuloside A	*Y* = 7632.32*X* + 1876.44	0.9998	0.0558–0.5533	0.8400
Echinacoside	*Y* = 391078*X* + 735, 276	0.9999	0.0100–0.0998	0.0022
2′-Acetylacteoside	*Y* = 2399320*X* + 3719.27	0.9995	0.0031–0.0441	0.1950
Acteoside	*Y* = 1441030*X* + 1983.61	0.9995	0.0119–0.1075	0.0132
Isoacteoside	*Y* = 2460430*X* − 282.725	0.9996	0.0015–0.0150	0.0152
Cistanoside F	*Y* = 1978120*X* − 158.191	0.9999	0.0009–0.0088	0.0102
Salidroside	*Y* = 942200X − 56.6313	0.9997	0.0005–0.0041	0.0013
Geniposidic acid	*Y* = 460926*X* + 86.9228	0.9996	0.0009–0.0095	0.0224
Ajugol	*Y* = 12080.3*X* + 72.2771	0.9997	0.0057–0.0589	0.0013
Catalpol	*Y* = 354984*X* − 122.491	0.9996	0.0047–0.0485	0.0598

**Table 3:** Upstream and downstream substrate of CaM.

	CaM	*β*-Actin
Upstream primers	5′-GATAAGGACGGCAATGGCTAC-A-3′	5′-CCT-GTGGCATCCATGAAACTAC-3′
Downstream primers	5′-CGATGTCTGCTTCCCTGATCAT-3′	5′-CTTCTG-CATCCTGTCAGCGAT-3′
Amplification fragments	115 bp	134 bp

**Table 4:** Content of 10 components in CD and WCD extract.

Compounds	CD (mg/g)	WCD (mg/g)
Tubuloside A	0.1720 ± 0.0548	0.2833 ± 0.0005
Echinacoside	1.2180 ± 0.4425	5.5261 ± 0.3468
2′-Acetylacteoside	0.6830 ± 0.2622	0.6991 ± 0.0188
Acteoside	1.3058 ± 0.4567	2.5991 ± 0.0576
Isoacteoside	0.2628 ± 0.0929	0.5980 ± 0.0186
Cistanoside F	0.4477 ± 0.1484	0.3992 ± 0.0099
Salidroside	0.2028 ± 0.0522	0.0928 ± 0.0027
Geniposidic acid	0.2369 ± 0.0766	0.0779 ± 0.0029
Ajugol	0.5011 ± 0.0134	0.3114 ± 0.0250
Catalpol	0.1509 ± 0.0353	0.0894 ± 0.0048

## Data Availability

The data used to support the findings of this study are included within the article.
